# Joint symptoms associated with anastrozole and letrozole in patients with breast cancer: a retrospective comparative study

**DOI:** 10.1186/s40780-017-0095-6

**Published:** 2017-11-07

**Authors:** Yoshihito Morimoto, Shuhei Sarumaru, Yuko Oshima, Chiho Tsuruta, Kazuhiro Watanabe

**Affiliations:** 1 Education and Research Center for Clinical Pharmacy, Showa Pharmaceutical University, 3-3165 Higashi-Tamagawagakuen, Machida, Tokyo, 194-8543 Japan; 2 Breast Clinic Tsukiji, Kaken Tsukiji Building 4F, 11-6 Akashi-cho, Chuo-ku, Tokyo, 104-0044 Japan

**Keywords:** Anastrozole, Aromatase inhibitors, Breast cancer, Joint symptoms, Letrozole

## Abstract

**Background:**

Joint symptoms are a common side effect of aromatase inhibitors. However, it is not known if the risk of these symptoms varies between the members of this drug class. The aim of this study was to compare the frequency of joint symptoms associated with anastrozole and that associated with letrozole.

**Methods:**

We retrospectively reviewed patients with breast cancer who were treated with anastrozole or letrozole at Tsukiji Breast Clinic, Japan, between April 2008 and July 2014. Joint symptoms were deemed to include both joint pain and painless joint symptoms. The time to onset of joint symptoms in the anastrozole group was compared with that in the letrozole group using Kaplan–Meier curves and the log-rank test.

**Results:**

Of 141 patients identified to have received aromatase inhibitors, 70 had been treated with anastrozole and 71 with letrozole. Joint symptoms occurred in 60.3% of the 141 patients (60.0% in the anastrozole group and 60.6% in the letrozole group; *p* = 1). Median time to appearance of joint symptoms was 583 days, with no significant difference between the anastrozole and letrozole groups (*p* = 0.962). There was no significant difference in time to onset of joint pain (*p* = 0.139); however, time to onset of painless joint symptoms was significantly shorter in the anastrozole group (*p* = 0.022). The sites at which joint symptoms occurred were similar in the two groups.

**Conclusions:**

The results of this study indicate that there is no difference in the pattern of occurrence of joint symptoms caused by anastrozole and those caused by letrozole.

**Trial registration:**

Trial registration was not required for this study because of its retrospective nature and lack of intervention.

## Background

Aromatase inhibitors are frequently used to treat postmenopausal women with hormone receptor-positive breast cancer. Anastrozole has demonstrable efficacy compared with tamoxifen as adjuvant therapy for postmenopausal women with this disease [1]. The Breast International Group (BIG) 1–98 study also reported that letrozole is effective and safe when used as adjuvant monotherapy [2]. Furthermore, a meta-analysis showed that aromatase inhibitors improve overall survival in postmenopausal women with hormone receptor-positive advanced breast cancer [3]. Therefore, aromatase inhibitors are recommended as primary endocrine therapy for these women. However, these agents are associated with a high frequency of joint symptoms. The ATAC trial showed that the frequency of arthralgia attributable to anastrozole was 35.6% [4]. In the BIG 1–98 study, letrozole caused arthralgia in 20.0% of patients [5]. Furthermore, in a Japanese study, joint pain and stiffness occurred in 61.6 and 59.2%, respectively, of women treated with anastrozole [6]. However, although it is known that aromatase inhibitors are associated with a high rate of joint symptoms, comparative data for the agents in this class are limited. The incidence of joint symptoms may differ between anastrozole and letrozole.

The aim of this study was to compare the frequency, time to onset, and sites of joint symptoms associated with anastrozole and letrozole in patients with breast cancer.

## Methods

The study protocol was approved by the research ethics committee of Showa Pharmaceutical University (approval numbers 2011–17 and 2014–3) and conducted in accordance with the Declaration of Helsinki. The need for informed consent was waived in view of the retrospective and observational nature of the study. Patients who had been prescribed anastrozole or letrozole at the Tsukiji Breast Clinic between April 2008 and July 2014 were identified. Patients who made fewer than 10 visits to the clinic and those who had joint symptoms prior to administration of aromatase inhibitors were excluded, as were patients who had received combination chemotherapy.

### Evaluation

Data on patient characteristics and occurrence and site of joint symptoms were collected. Joint symptoms were defined as either joint pain or painless joint symptoms, such as joint stiffness and decreased joint motion. Joint symptoms were defined as joint pain or painless joint symptoms that occurred on at least one occasion. All the joint symptoms associated with aromatase inhibitors were evaluated during interviews conducted by the same doctor.

### Statistical analysis

The Mann–Whitney *U* test and Fisher’s exact test were used to identify any differences in patient characteristics between the anastrozole and letrozole groups. The frequency of joint symptoms was compared between the two treatment groups using Fisher’s exact test. Time to onset of joint symptoms was compared between the two groups using Kaplan–Meier curves and the log-rank test. The starting point of the Kaplan–Meier curves was the day on which an aromatase inhibitor was started. The statistical analysis was performed using EZR version 1.35 software (R Foundation for Statistical Computing, Vienna, Austria) [7]. A *p*-value < 0.05 was considered to be statistically significant.

## Results

### Patient characteristics

Data were obtained for 141 patients (anastrozole group, *n* = 70; letrozole group, *n* = 71). Patient characteristics are shown in Table 1. Significantly more women had a history of receiving hormone therapy in the letrozole group than in the anastrozole group (*p* < 0.01). All patients with a history of hormone therapy, except for one in the letrozole group, had received tamoxifen. Significantly more women in the letrozole group had a history of chemotherapy than in the anastrozole group (*p* = 0.029).

**Table 1:** Patient characteristics

	Anastrozole	Letrozole	*p* *-*value
Patients (n)	70	71	
Median age, years (range)	64 (51–84)	64 (49–88)	0.579^a^
Stage			
0	1	0	
I	20	15	
IIA	25	21	
IIB	6	14	
IIIA	5	8	
IIIB	5	4	
IIIC	2	3	
IV	1	1	
Recurrence	4	4	
Unknown	1	1	
History of hormone therapy			
Yes	10	24	< 0.01^b^
No	60	47	
History of chemotherapy			
Yes	27	41	0.029 ^b^
No	43	30	

^a^ Mann–Whitney *U* test

^b^Fisher’s exact test

### Frequency of joint symptoms

Table 2 shows the frequency of joint symptoms. The overall frequency of joint symptoms was 60.3% (60.0% in the anastrozole group versus 60.6% in the letrozole group; *p* = 1). Painless joint symptoms occurred significantly more often in the anastrozole group than in the letrozole group (41.4% vs. 23.9%; *p* = 0.032). However, there was no significant difference in occurrence of joint pain between the groups (*p* = 0.239).

**Table 2:** Frequency of joint symptoms

	Total	Anastrozole	Letrozole	*p*-value
Patients (n)	141	70	71	
Joint symptoms (%)	85 (60.3)	42 (60.0)	43 (60.6)	1^a^
Joint pain (%)	66 (46.8)	29 (41.4)	37 (52.1)	0.239^a^
Painless joint symptoms (%)	46 (32.6)	29 (41.4)	17 (23.9)	0.032^a^

^a^ Fisher’s exact test

### Time to onset of joint symptoms

**Fig. 1:**
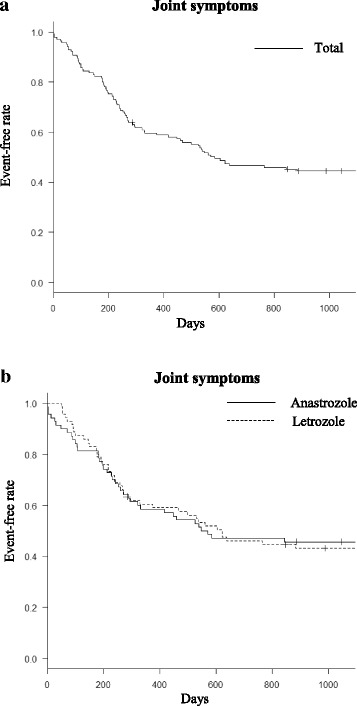
Event-free curves for joint symptoms. **a** Combined data and (**b**) data for anastrozole vs. letrozole. There was no statistically significant difference in time to onset of joint symptoms between the anastrozole and letrozole groups (*p* = 0.962)

**Fig. 2:**
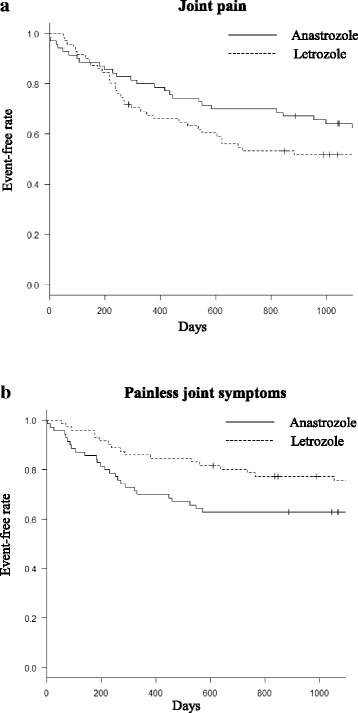
Event-free curves for (**a**) joint pain and (**b**) painless joint symptoms for anastrozole vs. letrozole. There was no statistically significant difference in occurrence of joint pain (**a**, *p* = 0.139, log-rank test). However, the time to onset of painless joint symptoms was significantly shorter in the anastrozole group than in the letrozole group (**b**, *p* = 0.022, log-rank test)

Figure 1a shows Kaplan–Meier curves for the overall rate of joint symptoms. Median time until occurrence of joint symptoms was 583 days. Figure 1b shows Kaplan–Meier curves for the rate of development of joint symptoms in the anastrozole and letrozole groups. Median time until occurrence of joint symptoms was not significantly different between the two groups (558.5 days in the anastrozole group and 621 days in the letrozole group; *p* = 0.962). Figure 2a, b shows the respective Kaplan–Meier curves for the rates of joint pain and painless joint symptoms. Time to onset of painless joint symptoms was significantly shorter in the anastrozole group than in the letrozole group (*p* = 0.022). However, there was no significant difference in time to onset of joint pain between the two groups (*p* = 0.139).

### Sites of joint symptoms

Table 3 shows the sites of joint pain and painless joint symptoms in the anastrozole and letrozole groups. Joint pain occurred at various sites in both groups, although the data were incomplete for the letrozole group. In both groups, the most frequently reported site of painless joint symptoms was the wrist/hand.

**Table 3:** Site of joint symptoms (joint pain and painless joint symptoms)

	Anastrozole	Letrozole
Joint pain		
Wrist/Hand	5	5
Elbow	0	0
Shoulder	5	8
Back	0	1
Hip	3	2
Knee	9	4
Feet	2	3
Total body	4	0
Unknown	1	14
Painless joint symptoms		
Wrist/Hand	15	15
Elbow	1	0
Shoulder	1	0
Back	2	1
Hip	0	0
Knee	3	0
Feet	0	3
Total body	1	1
Unknown	3	0

## Discussion

In this study, there was no difference in the frequency or time to onset of joint symptoms between women who received anastrozole and those who received letrozole. Aromatase inhibitor-related joint symptoms are known to be common. Yagata et al. reported a high rate of joint pain and stiffness associated with anastrozole [6], and a prospective multicenter cohort study in Japan reported a 71.8% rate of new or worsening joint symptoms in women receiving anastrozole [8]. Furthermore, Mao et al. reported rates of arthralgia associated with anastrozole, letrozole, and exemestane of 45, 49, and 48%, respectively, with no significant difference in the rates between treatment groups [9]. We also found a high rate of joint symptoms with anastrozole and letrozole (60.0 and 60.6%, respectively) and no significant difference between the groups. Ours is the first study to compare the rates and times to onset of joint symptoms between anastrozole and letrozole using Kaplan–Meier curves. There was no significant difference in time to onset of joint symptoms between the two groups; in most cases, joint symptoms occurred after more than 6 months of receiving aromatase inhibitor therapy, although symptoms did appear during the first 3 months of treatment in some cases. This finding suggests that aromatase inhibitor-related joint symptoms tend to develop late in treatment and should be watched for at this time. However, there have been reports of joint symptoms developing within the first 6 months of treatment [8, 9]. This discrepancy could be attributable to differences in background characteristics between the patients in the previous studies and those in our present study, and the fact that data were only collected for 1 year in the previous studies. Kanematsu et al. also reported that the onset of aromatase inhibitor-related arthralgia was sometimes delayed [10]. In the anastrozole group, the main sites of joint pain and painless joint symptoms were the knee and hand, respectively, as found in a previous study [6].

Anastrozole and letrozole improve disease-free survival in patients with breast cancer to a similar extent and have replaced tamoxifen as the adjuvant standard of care. However, anastrozole and letrozole differ in their structure, and both in vitro and animal models have shown differences in their mechanism and potency of estrogen suppression [11]. Geisler et al. identified letrozole to be a more potent suppressor of total body aromatization and plasma estrogen levels than anastrozole in postmenopausal women with metastatic breast cancer [12]. A head-to-head comparison (the Femara Versus Anastrozole Clinical Evaluation trial) is now under way to verify this difference in clinical effect [11]. In another study, there was no significant difference in quality of life scores between anastrozole and letrozole [13], but further evidence comparing these 2 drugs is awaited.

Although the exact mechanism of the joint symptoms associated with the aromatase inhibitors is still unclear, an aromatase inhibitor-induced decrease in estrogen levels might play an important role in the pathogenesis of these joint symptoms [14]. In our study, the rate of painless joint symptoms was significantly higher in the anastrozole group than in the letrozole group and the time to onset of these symptoms was significantly shorter in the anastrozole group than in the letrozole group. Joint pain tended to develop earlier in the letrozole group than in the anastrozole group, but the difference was not statistically significant. The reasons for these differences are unknown, but may lie in differences in the extent of suppression of total aromatization and plasma estrogen levels between anastrozole and letrozole. A prospective trial would be needed to confirm if such a difference exists.

Patient tolerability and compliance with aromatase inhibitor therapy is a very important issue because of the extended duration of treatment involved. In addition to causing joint symptoms, the side effects of aromatase inhibitors include hot flashes, osteoporosis, fractures, hypercholesterolemia, and cardiovascular events [11]. Joint symptoms adversely impact health-related quality of life in many patients and reduce compliance. We hope that our findings will help in selection of appropriate adjuvant therapy for women with breast cancer.

The present study has several limitations. First, it had a retrospective, single-center design and a small sample size. Furthermore, a significantly greater proportion of women in the letrozole group had a history of hormone therapy when compared with the anastrozole group. Previous hormone therapy is thought to be associated with joint symptoms [15, 16]. Similarly, a significantly greater number of women had a history of chemotherapy in the letrozole group than in the anastrozole group, so the patient background characteristics were not comparable. Taxane chemotherapy has been reported to contribute to expression of joint symptoms with aromatase inhibitors [14, 15]. Second, patients with breast cancer may also have symptoms of menopause, which can be difficult to distinguish from the side effects of aromatase inhibitors. We excluded patients with joint symptoms before administration of an aromatase inhibitor to eliminate this potential source of confounding as far as possible.

## Conclusion

No difference was observed in the rate of joint symptoms associated with administration of anastrozole and letrozole.
